# Hospitals That Serve Many Black Patients Have Lower Revenues and Profits: Structural Racism in Hospital Financing

**DOI:** 10.1007/s11606-022-07562-w

**Published:** 2022-08-05

**Authors:** Gracie Himmelstein, Joniqua N Ceasar, Kathryn EW Himmelstein

**Affiliations:** 1 Department of Medicine, University of California Los Angeles, Los Angeles, CA USA; 2 Departments of Medicine and Pediatrics, The Johns Hopkins Hospital and The Johns Hopkins University School of Medicine, Baltimore, MD USA; 3 Division of Infectious Diseases, Department of Medicine, Massachusetts General Hospital and Harvard Medical School, Boston, MA USA

**Keywords:** structural racism, hospital payment, Black-serving hospitals

## Abstract

**Background:**

Care for Black patients is concentrated at a relatively small proportion of all US hospitals. Some previous studies have documented quality deficits at Black-serving hospitals, which may be due to inequities in financial resources for care.

**Objective:**

To assess disparities in funding between hospitals associated with the proportion of Black patients that they serve.

**Participants:**

All Medicare-participating hospitals, 2016–2018.

**Main Measures:**

Patient care revenues and profits per patient day at Black-serving hospitals (the top 10% of hospitals ranked by the share of Black patients among all Medicare inpatients) and at other hospitals, unadjusted and adjusted for differences in case mix and hospital characteristics.

**Key Results:**

Among the 574 Black-serving hospitals, an average of 43.7% of Medicare inpatients were Black, vs. 5.2% at the 5,166 other hospitals. Black-serving hospitals were slightly larger, and were more often urban, teaching, and for-profit or government (vs. non-profit) owned. Patient care revenues and profits averaged $1,736 and $−17 per patient day respectively at Black-serving hospitals vs. $2,213 and $126 per patient day at other hospitals (*p*<.001 for both comparisons). Adjusted for patient case mix and hospital characteristics, mean revenues were $283 lower/patient day (*p*<.001) and mean profits were $111/patient day lower (*p*<.001) at Black-serving hospitals. Equalizing reimbursement levels would have required $14 billion in additional payments to Black-serving hospitals in 2018, a mean of approximately $26 million per Black-serving hospital.

**Conclusions:**

US hospital financing effectively assigns a lower dollar value to the care of Black patients. To reduce disparities in care, health financing reforms should eliminate the underpayment of hospitals serving a large share of Black patients.

**Supplementary Information:**

The online version contains supplementary material available at 10.1007/s11606-022-07562-w.

## Introduction

While Title VI of the 1964 Civil Rights Act forbade hospitals from discriminating based on race, *de facto* segregation has persisted. In 2004, 5% of hospitals cared for 44% of Black Medicare patients,^1^ and in 2010–2011 three-quarters of all Black infants in the USA were born in just one-quarter of US hospitals.^2^ While racism in non-medical spheres certainly contributes to stark racial differences in health outcomes—including the 5.8 year gap in life expectancy between Black and White Americans^3^—inequities in medical care are also important. Numerous studies have documented racial disparities, including in obstetrical and surgical outcomes, rates of avoidable hospitalizations among seniors, and adherence to guidelines for care of patients with common emergencies such as acute myocardial infarction.^2,4–7^ While some studies suggest that White and Black patients receive similar quality care within a given hospital, Black patients are more likely to receive care at facilities with lower scores on quality metrics.^2,8–11^

Moreover, hospitals where Black patients account for a large share of inpatients have relatively modest facilities (as measured by the dollar value of their buildings and equipment), and they are less likely than other hospitals to offer some lucrative higher-tech services like cardiac catheterization labs.^12^ These asset deficits reflect longstanding disparities in funding: a decades- and even centuries-long paucity of donations, government subsidies, and operating surpluses to finance hospital construction in Black neighborhoods.

We sought to assess whether current financing patterns ameliorate or reproduce resource inequities. Using financial data submitted annually to Medicare by nearly all US hospitals, we compared the revenues and profits of the ten percent of hospitals with the highest shares of Black inpatients (hereinafter “Black-serving hospitals”) to other US hospitals.

## Materials and Methods

This study, which did not use or produce any identifiable private data, is not considered human subjects research under Princeton University Institutional Review Board guidelines.

### Data Sources

We used data on Medicare fee-for-service inpatients from 2016 Medicare Part A “100% files” to determine the racial composition of each Medicare-participating hospital’s inpatients. Using each hospital’s unique Medicare Provider Number, we linked the racial mix data to 2016–2018 Medicare Cost Reports downloaded from CMS’ Healthcare Cost Report Information System,^13^ 2016–2018 American Hospital Association (AHA) Survey data,^14^ and the 2017 Medicare Case Mix Index (CMI) file.^15^

Medicare Cost Reports, which include data on hospitals’ characteristics and finances, are submitted annually to CMS by all Medicare-participating hospitals and are subject to government audit.

We used the CMI to adjust for differences in patient mix. The CMI is the average weight of all diagnosis-related group (DRG) claims submitted to Medicare during the fiscal year by each hospital, excluding those (e.g., Critical Access Hospitals) that are not paid based on DRGs. DRGs are designed to assess the resources (and expected cost) required to care for a patient with a particular diagnosis. Hence, the CMI is a summary measure of the influence of a hospital’s case mix (i.e., the type of patients the hospitals treats, and the severity of their medical conditions) on the expected costs of care for its Medicare-insured patients.

We assessed hospitals’ revenues, costs, and profits per unit of service (i.e., per inpatient day). Because most of the financial data reported in the Medicare Cost Reports encompasses both inpatient and outpatient services, our analyses used AHA figures for adjusted inpatient days as the denominator for our estimates. The AHA calculates adjusted inpatient days according to the formula:

Adjusted Inpatient Days = Inpatient Days + (Inpatient Days × (Outpatient Revenue/Inpatient Revenue)).

Additionally, to assess the robustness of our assumption that the racial mix and complexity of Medicare-insured inpatients is representative of all inpatients, we analyzed the 2017 National Inpatient Sample (NIS), an all-payer database.^16^ The NIS includes hospital-supplied data on patient race (available for 96.1% of discharges) and hospital charges, an indicator of patients’ relative complexity (and hospital resource use) for most hospitals in 48 states. However, we could not use the NIS for our main analyses because it lacks key variables (including hospital profits, and revenues from government subsidies, investments, and contributions) and cannot be linked to the Medicare or AHA data.

### Statistical Analysis

We ranked hospitals by the share of Black patients among their Medicare (fee-for-service) inpatients and categorized the 10% of hospitals with the highest shares (>26.8%) of Black patients as Black-serving, as has been done in previous studies.^1,12^

Using the Medicare Cost Reports we determined, for each hospital, average annual patient care revenues and total profit (often referred to as total surplus in non-profit or government-owned facilities) from all sources, including investment and rental income, donations, and government subsidies. We first removed part-year and duplicate reports and extreme outliers (the top and bottom 1% of each calculated financial measure), which likely represent decimal point or other reporting errors in the administrative data. We then calculated our main outcome measures: mean and median revenues and profits per adjusted inpatient day (hereinafter “per patient day”) for Black-serving vs. other hospitals. We used *t*-tests to assess differences in means, and Wilcoxon Rank Sum tests for differences in medians.

Because hospitals’ costs are affected by the case mix and other hospital characteristics, we used multivariable linear regression to calculate inequities in revenues and profits adjusted for differences between Black-serving and other hospitals. These models adjusted for teaching status, urban/rural location, census region, ownership (for-profit, public, or non-profit), size (annual number of discharges), and each hospital’s Medicare CMI.

We then estimated the total amount of new revenues that would be needed to bring revenues for Black-serving hospitals to parity with other US hospitals by multiplying the adjusted difference in revenues per patient day by the total number of adjusted inpatient days in Black-serving hospitals.

To explore why Black-serving hospitals had lower revenues and profits, we performed two sets of multivariable analyses that included the predictor variables in the main models described above, plus additional adjustment for indicators of payer mix derived from Medicare Cost Reports. The first sets of models added adjustment for Medicaid discharges as a share of all discharges. The second set added adjustment for the ratio of total patient care revenues to “unreimbursed and uncompensated” care, a measure that includes revenue shortfalls attributable to Medicaid underpayments, and is generally considered less reliable than other figures in the Cost Reports.

We then performed three sensitivity analyses: (1) using unadjusted inpatient days; (2) adjusting for individual states rather than census region; and (3) without trimming the top and bottom 1% of calculated financial measures.

Finally, to test whether the racial mix and case mix of Medicare inpatients is representative of all inpatients, we used the NIS to assess the correlations between the proportions of Black Medicare inpatients and all inpatients, and between the charges incurred by Medicare inpatients and all inpatients at each hospital using Pearson correlation coefficients.

All analyses were carried out using SAS version 9.4.

## Results

Our sample included 574 Black-serving and 5,166 other US hospitals. At Black-serving hospitals, an average of 43.7% of inpatients were Black vs. 5.2% at other hospitals; Medicaid discharges accounted for 14.2% of all discharges at Black-serving hospitals vs. 9.5% at others. Consistent with population patterns, more Black-serving hospitals were located in the South and fewer were in the West. Black-serving hospitals were slightly larger (as measured by annual patient discharges), and were more often urban, teaching, and for-profit or government (vs. non-profit) (Table 1).

**Table 1:** Characteristics of Black-serving and other US hospitals, 2016–2018

	**Black-serving hospitals** **(*n*** **= 574)**	**Other hospitals** **(*n*= 5,166)**	*p*-value for difference*
**Hospital characteristics**			
Black patient’s share of all inpatients, *Mean*	43.7%	5.2%	<.001
Discharges/year, 2016–2018, *Median (IQR)*	2,006 (504–9,136)	1,867 (497–7,761)	.16
Medicaid discharges as % of total discharges	14.2%	9.5%	<.001
Teaching hospital, *% of hospitals*	34.5%	21.6%	<.001
Census region*, % of hospitals*			<.001
Northeast	10.5%	13.0%	
South	67.5%	37.0%	
Midwest	17.9%	29.8%	
West	4.2%	20.2%	
Urban location, *% of hospitals*	74.8%	59.6%	<.001
Ownership			.<001
Non-profit	37.1%	52.1%	
For-profit	32.3%	26.1%	
Government	30.6%	21.9%	
Case Mix Index*, 2017, *Median (IQR)*	1.53 (1.34–1.76)	1.59 (1.42–1.81)	.005

Source: Authors’ analysis of CMS data

*Medicare Case Mix Index is the average DRG weight of all Medicare inpatients served in FY 2017 as computed by CMS and published in its 2019 Final Rule

*IQR* inter-quartile range

**Fig. 1:**
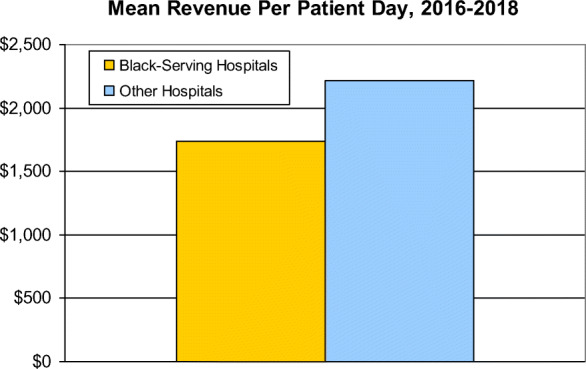
Mean revenue per patient day, 2016–2018

Total reimbursements (including payments by insurers and patients) for patient care per day averaged 21.6% lower at Black-serving than at other hospitals ($1,736 vs. $2,213) (Table 2 and Figure 1). Other hospitals garnered slightly more funds from investments ($13.67 vs. $6.22 per patient day, *p*<.001) and contributions ($6.84 vs. $5.45 per patient day, *p*<.001), while the level of government subsidies did not differ.

**Table 2:** Financial performance of Black-serving and other US hospitals, 2016–2018

	**Black-serving hospitals** **(*n*** **= 541)**	**Other hospitals** **(*n*= 4,969)**	***p*-value for unadjusted difference in means**	Adjusted difference in means*	***p*-value for adjusted difference in means**
**Reimbursement for patient care per adjusted patient day**					
Mean	$1,736	$2,213	.0001	-$283	<.001
Median [IQR]	$1,599 [$527−$2,225]	$1,972 [$1,200, $2,859]	NA	NA	NA
**Total profit/surplus per adjusted patient day**					
Mean	-$17	$126	.0001	-$111	<.001
Median [IQR]	$8 [−$76, +$117]	$64 [−$19, +$233]	NA	NA	NA

Source: Authors’ analysis of CMS data

*Linear regression models adjusted for annual hospital discharges, teaching status, census region (Northeast, Midwest, South, West), urban location, ownership (non-profit, for-profit, government) and Medicare Case Mix Index

*IQR* inter-quartile range

The hospitals’ bottom lines differed significantly. After accounting for income from patient care and all other sources (and subtracting hospitals’ costs), profit/surplus per patient day at Black-serving hospitals was small (a median profit of $8), or negative (a mean loss of $17). Median and mean profits/surpluses at other hospitals were $64 and $126 per patient day respectively (Table 2).

**Fig. 2:**
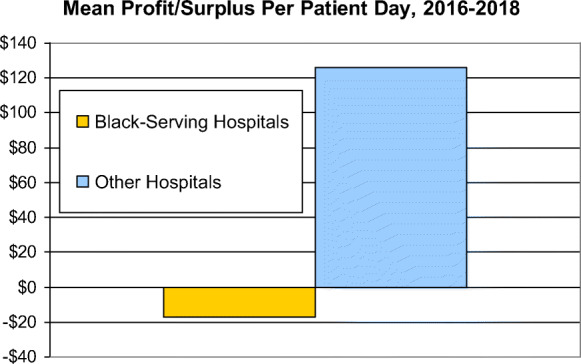
Mean profit/surplus per patient day, 2016–2018

In multivariable analyses that controlled for hospital characteristics and the complexity of care their patients required, Black-serving hospitals had $283 lower reimbursements per patient day than other hospitals and $111 lower total profit/surplus (Table 2 and Figure 2 ). These figures imply that equalizing reimbursement levels would have boosted Black-serving hospitals’ revenues by about $14 billion in 2018, equivalent to approximately $26 million per Black-serving hospital.

In multivariable models that added adjustment for Medicaid’s share of discharges, or for uncompensated/unreimbursed care relative to total revenues, Black-serving hospitals’ relative disadvantage in revenues (but not their disadvantage in profit/surplus) was reduced by about one-third (Appendix Tables 3 and 4), suggesting that payer mix accounted for some, but not all of the financial disparities we observed.

The sensitivity analysis adjusted for state rather than region yielded similar results to our main models; Black-serving hospitals had $257 lower reimbursements per patient day than other hospitals and $134 lower profits/surpluses (*p*<.001 for both figures). Analyses using actual inpatient days (rather than adjusted inpatient days) yielded substantially larger estimates of Black-serving hospitals’ financial disadvantage (Appendix Table 5). Analyses that included the extreme outliers that were trimmed for our main analyses were numerically similar to our main results, although *p* values were larger, as expected with the inclusion of some erroneous values that introduce statistical noise (Appendix Table 6).

In the NIS data, Black patients’ share of Medicare discharges was closely correlated with their share of all discharges (*r*=0.97, *p*<.001), and charges for Medicare inpatients were closely correlated with charges for all inpatients (*r*=0.95, *p*<.001)—supporting our use of the racial mix and case mix of Medicare inpatients as proxies for hospitals’ overall racial mix and case mix.

## Discussion

Our findings indicate that hospitals that serve many Black patients receive lower payments for patient care. They also accrue lower profits/surpluses than other hospitals, although these differences are somewhat smaller, suggesting that Black-serving hospitals have a leaner cost structure.

These ongoing funding disparities reinforce inequities in resources inherited from the past, and may undermine the quality of care at Black-serving hospitals; Black-serving hospitals score lower on some quality indicators, have lower nurse staffing ratios,^1^ and often lack important services.^12^ In some cases, financial losses have forced the closure of Black-serving hospitals, a fate that has befallen several in Philadelphia,^17^ including Hahnemann University Hospital (where our Medicare racial mix data indicates that about half of patients were Black). Recently, the COVID-19 pandemic has called attention to the stark inequities in hospitals’ facilities.^18^

Our multivariable models with added adjustment for Medicaid and unreimbursed/uncompensated care provide evidence that differences in insurance coverage underlie some of the funding differences we observed. A larger proportion of Black than White individuals are uninsured or covered by Medicaid, which generally provides lower payments to hospitals as compared to private insurance or Medicare. Even among employed persons, Black workers are less likely than White workers to have job-based private coverage.^19^ Moreover, among those with Medicare or private insurance, Black patients’ lower incomes and assets^20^ may leave them less able to pay the often-substantial deductibles or coinsurance for inpatient care.

Medicaid patients’ second-class status was baked in at the program’s outset; in the midst of the Civil Rights era, the Congress chose to separate coverage for the poor (many of whom were Black) from that of the elderly (most of whom were White). Medicare offered seniors a federal plan modeled on Blue Cross coverage, while Medicaid, passed simultaneously, relegated the poor to a welfare-based program largely controlled by state governments, some of them openly racist. Even as the Affordable Care Act expanded coverage, its heavy reliance on Medicaid perpetuated the racial coverage divide.

### Limitations

Although our analysis of NIS data indicates that the racial mix and case mix of each hospital’s Medicare patients is representative of its overall inpatient population, some error is likely. While the Medicare data accurately identifies Black race/ethnicity, its designation of Hispanics, Asian/Pacific Islanders, and especially Native Americans is often erroneous,^21^ obstructing identification of hospitals disproportionately serving members of those groups. Our data compare Black-serving to other US hospitals and may not apply to all safety-net hospitals, many of which primarily serve non-Black patients.

Adjusting for differences in patient factors that affect each hospital’s costs of care is complex. While Medicare’s Case Mix Index, the measure used in our multivariable models, is specifically designed to predict the costs associated with care of the average patient at each hospital, it does so imperfectly. For instance, hospitals generally find elective orthopedic and cardiac procedures highly profitable, suggesting that the Medicare Case Mix Index may overestimate the resources required to perform them.

## Conclusion and Implications

Nearly two decades ago, an Institute of Medicine report documented the health harms of structural racism in health care, noting that “financial and institutional arrangements of health systems . . . and [the] policy environment in which they operate, may have disparate and negative effects on minorities’ ability to attain quality care.”^22^ Little progress has occurred since then, a point reinforced by our findings.^23–25^

Our multi-tiered health insurance system continues to assign a lower dollar value to the care of Black patients. That system embodies the legacies of slavery and discrimination, the persistence of institutional traditions, and health care financing policies that perpetuate Black people’s disadvantage.

Legal definitions of discrimination codified during the Civil Rights era include both disparate treatment and disparate impact. Disparate treatment requires evidence of racist intent. Disparate impact acknowledges that apparently race-neutral policies may nonetheless be discriminatory. Our findings do not speak to the intentions of present-day policy and health care leaders. However, we find evidence of disparate impact, indicating that the current system of hospital financing is a form of structural racism.

Health financing reforms should assure that hospital payment reflects patients’ care needs rather than their race, and repair the damage of past policies by preferentially directing new capital funds to resource-starved facilities that have long served Black communities.

## Supplementary Information

ESM 1 (DOCX 30 kb)

## Declarations

### Disclaimer

The content is solely the responsibility of the authors and does not reflect the views of the NIH.
