# Supplementary material for: Hospitals That Serve Many Black Patients Have Lower Revenues and Profits: Structural Racism in Hospital Financing
Source: J Gen Intern Med. 2022 Aug 5;38(3):586–91. doi: 10.1007/s11606-022-07562-w (PMC9361904; doi:10.1007/s11606-022-07562-w)
Supplement: Supplementary file 1 — (DOCX 30 kb) [file 11606_2022_7562_MOESM1_ESM.docx]

1. Appendix

Table 3 Financial performance of Black-serving and other US hospitals, with additional adjustment for Medicaid discharges as a share of total discharges, 2016–2018

|  | **Adjusted difference between Black-serving and other hospitals**[*****](#TF1) | ***p*-value for adjusted difference in means** |
| --- | --- | --- |
| **Reimbursement for patient care per adjusted patient day** | −$173 | .02 |
| **Total profit/surplus per adjusted patient day** | −$122 | <.001 |

Source: Authors’ analysis of CMS data

*Linear regression models adjusted for annual hospital discharges, teaching status, census region (Northeast, Midwest, South, West), urban location, ownership (non-profit, for-profit, government), Medicare Case Mix Index and Medicaid discharges as a share of total discharges

Table 4 Financial performance of Black-serving and other US hospitals, with additional adjustment for the ratio of total patient revenues to unreimbursed/uncompensated care, 2016–2018

|  | **Adjusted difference between Black-serving and other hospitals**[*****](#TF2) | ***p*-value for adjusted difference in means** |
| --- | --- | --- |
| **Reimbursement for patient care per adjusted patient day** | −$205 | .005 |
| **Total profit/surplus per adjusted patient day** | −$111 | <.001 |

Source: Authors’ analysis of CMS data

*Linear regression models adjusted for annual hospital discharges, teaching status, census region (Northeast, Midwest, South, West), urban location, ownership (non-profit, for-profit, government), Medicare Case Mix Index and the ratio of total revenues to uncompensated/unreimbursed care

Table 5

Table 5 Sensitivity analysis. Financial performance of Black-serving and other US hospitals per actual (rather than adjusted) inpatient day, 2016–2018

|  | **Black-serving hospitals** | **Other hospitals** | ***p*-value for unadjusted difference in means** | **Adjusted difference in means**[*****](#TF3) | ***p*-value for adjusted difference in means** |
| --- | --- | --- | --- | --- | --- |
| **Reimbursement for patient care per inpatient day** |  |  |  |  |  |
| Mean | $7,317 | $13,415 | <.001 | −$1139 | .02 |
| Median [IQR] | $5,569  [1,862, 8,239] | $8,737  [$5,153, $16,823] | NA | NA | NA |
| **Total profit/surplus per inpatient day** | | | | | |
| Mean | −$170 | $418 | <.001 | −$445 | .002 |
| Median [IQR] | $12  [−$309, +$242] | $239  [−$113, + $927] | NA | NA | NA |

Source: Authors’ analysis of CMS data

*Linear regression models adjusted for annual hospital discharges, teaching status, census region (Northeast, Midwest, South, West), urban location, ownership (non-profit, for-profit, government) and Medicare Case-mix Index

*IQR* inter-quartile range

Table 6

Table 6 Sensitivity analysis. Financial performance of Black-serving and other US hospitals per adjusted inpatient day, without trimming of top and bottom 1%, 2016–2018

|  | **Black-serving hospitals** | **Other hospitals** | ***p*-value for unadjusted difference in means** | **Adjusted difference in means**[*****](#TF4) | ***p*-value for adjusted difference in means** |
| --- | --- | --- | --- | --- | --- |
| **Reimbursement for patient care per inpatient day** | | | | | |
| Mean | $1,758 | $2,472 | <.001 | −$239 | .03 |
| Median [IQR] | $1,617  [$932, $2,261] | $2,009  [$1,233, $2,920] | NA | NA | NA |
| **Total profit/surplus per inpatient day** | | | | | |
| Mean | −$133 | $90 | <.001 | −$144 | .12 |
| Median [IQR] | $8  [−$87, $1,129] | $68  [−$18, $240] | NA | NA | NA |

Source: Authors’ analysis of CMS data

*Linear regression models adjusted for annual hospital discharges, teaching status, census region (Northeast, Midwest, South, West), urban location, ownership (non-profit, for-profit, government) and Medicare Case-mix Index

*IQR* inter-quartile range
